# Natural selection drives leaf divergence in experimental populations of *Senecio lautus* under natural conditions

**DOI:** 10.1002/ece3.5263

**Published:** 2019-06-11

**Authors:** Thomas J. Richards, Daniel Ortiz‐Barrientos, Katrina McGuigan

**Affiliations:** ^1^ School of Biological Sciences St Lucia University of Queensland St Lucia Queensland Australia; ^2^ Department of Plant Biology Swedish University of Agricultural Sciences Linnean Center for Plant Biology Uppsala Sweden

**Keywords:** adaptation, divergent selection, LAMINA, Local adaptation, morphometrics, phenotype, reciprocal transplant, Senecio

## Abstract

1. Leaf morphology is highly variable both within and between plant species. This study employs a combination of common garden and reciprocal transplant experiments to determine whether differences in leaf shape between *Senecio lautus* ecotypes has evolved as an adaptive response to divergent ecological conditions.
2. We created a synthetic population of hybrid genotypes to segregate morphological variation between three ecotypes and performed reciprocal transplants where this hybrid population was transplanted into the three adjacent native environments. We measured nine leaf morphology traits across the experimental and natural populations at these sites.
3. We found significant divergence in multivariate leaf morphology toward the native character in each environment, suggesting environmental conditions at each site exert selective pressure that results in a phenotypic shift toward the local phenotype of the wild populations.
4. These associations suggest that differences in leaf morphology between *S. lautus* ecotypes have arisen as a result of divergent selection on leaf shape or associated traits that confer an adaptive advantage in each environment, which has led to the formation of morphologically distinct ecotypes.

## 1 INTRODUCTION

Spatial variation in natural selection can lead to phenotypic divergence within a species as local populations adjust to the prevailing environmental conditions (Niinemets, 2001). Adaptation can lead to reduced phenotypic and genetic variance around a particular phenotype that confers greatest fitness, leading to the formation of distinct ecotypes associated with local environmental conditions (Clausen, Keck, & Hiesey, 1941; Lowry, 2012; Turrill, 1946). Adaptation to local conditions is common in plants (Hereford, 2009) and is associated with clear mechanisms of reproductive isolation such as immigrant inviability (Baack, Melo, Rieseberg, & Ortiz‐Barrientos, 2015; Hendry, 2004) and assortative mating as a result of phenological (Lowry, Rockwood, & Willis, 2008; Savolainen et al., 2006) or phenotypic divergence (Stelkens & Seehausen, 2009). As such, it is likely that local adaptation plays a significant role in evolutionary diversification in the plant kingdom (Bomblies, 2010).

Of the many functional traits that confer adaptation to environmental conditions, leaves form a particularly variable aspect of plant architecture. Leaves are complex organs fundamental to physiological processes such as gas exchange and energy capture (Nicotra, Cosgrove, Cowling, Schlichting, & Jones, 2008) and are highly variable both within and between species (Andersson, 1991; Gurevitch, 1992; Wyatt & Antonovics, 1981). There is a range of evidence to suggest that leaf shape variation is subject to natural selection (reviewed in Givnish, 1979; Geber & Griffen, 2003; Chitwood & Sinha, 2016), and there are well‐documented associations between leaf shape and climate across many species (Givnish, 1987; Nicotra et al., 2011; Niinemets, 2001) suggesting a functional, and therefore potentially adaptive significance to many aspects of leaf size and shape.

An important consideration in the assessment of local adaptation is whether phenotypic differences between ecotypes can be attributed to plastic responses or heritable genetic variation between populations (Kawecki & Ebert, 2004; Sultan, 1995). Phenotypic plasticity is ubiquitous in plants (Palacio‐López, Beckage, Scheiner, & Molofsky, 2015) and can allow individuals to survive in unfavorable conditions (Chevin & Lande, 2011). However, evolutionary divergence and diversification requires some heritable variation underpinning phenotypic differences between ecotypes (Manier, Seyler, & Arnold, 2007). The evolutionary significance of phenotypic differentiation between ecotypes depends on whether traits have both a heritable genetic basis and are subject to divergent selection between environments (Abbott & Comes, 2007; Lowry, 2012).

A number of experimental approaches have been applied to determine whether traits are heritable, and subject to divergent selection between sites. Common garden trials are a powerful approach to distinguish between plastic versus heritable phenotypic differences between ecotypes (Kawecki & Ebert, 2004). Hybridization can be used to artificially segregate variation in the traits that differentiate ecotypes, and as such can be used to generate novel phenotypes that are not currently present in natural populations (Lexer, Randell, & Rieseberg, 2003; Schluter, 2000). Reciprocal transplant experiments under field conditions are used to compare the relative fitness of local and nonlocal phenotypes to determine the extent of local adaptation (Ågren & Schemske, 2012). Together, hybridization and field transplant experiments can be an effective tool to recreate the divergent response to selection that has potentially led to the emergence of observed phenotypic differences between populations and therefore the formation of ecotypes (Nagy, 1997).

Here, we use a combination of common garden‐ and field‐based experiments to determine how divergent natural selection shape patterns of leaf shape variation between ecotypes of a native Australian plant, *Senecio lautus*. Previous studies in this species complex have identified a number of ecotypes associated with specific environmental conditions (Radford, Cousens, & Michael, 2004) and have determined that adaptation to divergent environments is the major factor determining patterns of genetic differentiation (Roda, Liu, et al., 2013) and reproductive isolation within the species complex (Richards & Ortiz‐Barrientos, 2016).

We build on these prior findings to specifically test whether phenotypic differences in leaf shape between *S. lautus* ecotypes in the wild are heritable, and the result of ecologically based divergent natural selection. We quantify variation in leaf shape between three parapatric populations in the wild and then test whether variation in these divergent traits is heritable using common garden experiments. We then conduct a reciprocal transplant experiment using synthetic hybrid crosses in the three natural environments to determine whether ecological selection drives leaf shape divergence between experimental populations. Altogether, our results suggest that differences in leaf morphology between ecotypes of *S. lautus* are the product of ecologically based divergent natural selection.

## 2 MATERIALS AND METHODS

### 2.1 Study system

To determine the link between local adaptation and leaf phenotypes, we investigate patterns of leaf shape variation between three ecotypes within the *Senecio lautus* species complex (see Roda, Liu, et al., 2013 for discussion of current taxonomy). *Senecio lautus is* a short‐lived, outcrossing native plant with a distribution across southern Australia (Ali, 1964). The species complex consists of multiple distinct ecotypes (Radford et al., 2004) that have evolved repeatedly in response to similar ecological conditions (Roda, Ambrose, et al., 2013; Roda, Liu, et al., 2013). Plant morphology, including leaf shape, plant architecture, and growth habit, is maintained in common garden conditions suggesting phenotypic differences between ecotypes are genetically based (Ali, 1966; Ornduff, 1964; Radford et al., 2004). Ecologically based divergent selection has been shown to maintain distinction between ecotypes in the wild (Melo, Grealy, Brittain, Walter, & Ortiz‐Barrientos, 2014; Richards, Walter, McGuigan, & Ortiz‐Barrientos, 2016) as intrinsic genetic barriers to reproduction are low (Richards & Ortiz‐Barrientos, 2016). There is genetic variation for a range of adaptive characters within the system (Walter, Aguirre, Blows, & Ortiz‐Barrientos, 2018), suggesting that divergence in these characters could evolve in response to divergent ecological selection between populations.

**Figure 1:**
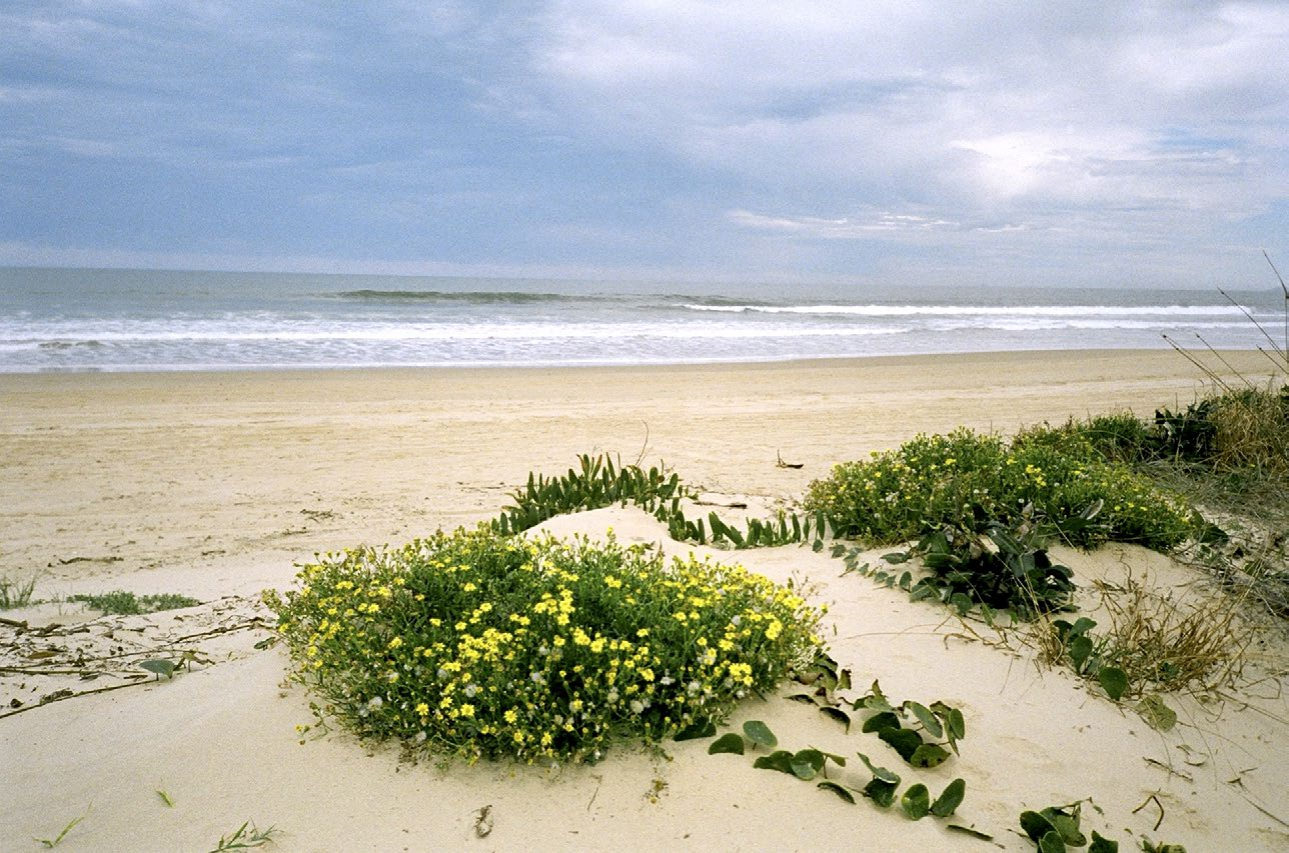
Dune Ecotype of Senecio lautus at Boambee beach, Coffs Harbour

Plants included in this work were measured in, or collected from, natural populations of three ecotypes of *Senecio lautus* at Boambee Beach (S 30°18'45.28", E 153°8'21.43", Dune type), Corambirra Point (S 30°18'44.09", E 153°8'41.51", Headland type), and Mutton Bird Island (S 30°18'19.67", E 153°8’57.27", Island type) at Coffs Harbour, NSW. These populations have been the subject of investigations into the importance of local adaptation in reproductive isolation (Richards & Ortiz‐Barrientos, 2016) and tests of the ecological speciation hypothesis (Richards et al., 2016). This previous work has identified strong patterns of differential establishment and growth across the three environments depending on the proportion of local genes carried. This demonstration of local adaptation provides an ideal setting to explore the impact of divergent ecological selection on phenotype divergence. These study locations provide an ideal experimental setting for testing the implications of divergent natural selection as natural populations occupying the three contrasting environments fall within ~800 meter radius, with the Dune and Headland populations are separated by ~10 m (Figure 1).

### 2.2 Leaf processing and data collection

Mature leaves were used in all aspects of this study to control for differences associated with heterophylly. Leaves were collected, pressed until dry, and then laminated. We included five leaves for all plants for which five leaves were available, individuals with less than three available mature leaves were removed from the analysis. Leaves were scanned using a Canoscan 9000i flatbed scanner at 300 dpi, and leaf shape analysis was conducted on these images using the morphometric software program Lamina BATCH (Bylesjo et al., 2008) to extract leaf shape data. Leaf shape is a complex trait which is described by multiple correlated morphological measurements. As such, we included nine widely used measures of leaf blade size and shape: area, margin perimeter, compactness, circularity, leaf width, leaf length, number of indents, indent density, and dissection (see Table S1). Mean trait values were calculated for each individual plant. By analyzing these measures in a multivariate framework, we can account for correlation between traits and investigate how different combinations of traits describe leaf shape divergence between environments.

### 2.3 Common garden experiment

#### 2.3.1 Genetic basis of traits

*Senecio lautus* seeds were collected from Headland, Dune and Island populations at Coffs Harbour, NSW. Two seeds each from 20 Dune, Headland, and Island families were scarified and germinated on moist filter paper in 40 mm petri dishes. Wild seeds were collected from plants separated by at least 15 m which reduces the probability of sampling siblings or closely related individuals. Seedlings were planted into a 50:50 sand–peat commercial potting media in 90 mm plastic pots and were grown in The University of Queensland Glasshouses in a randomized design under natural light and evaporative cooling. Plants were harvested at the end of the flowering period at 5 months of age. Plants that either failed to establish or perished before measurement were discarded from this analysis, leaving phenotypic measurements from 28 Dune, 37 Headland, and 22 Island individuals grown in common garden conditions. To determine whether there was heritable divergence in leaf morphology among the ecotypes, we tested the following multivariate analysis of variance (MANOVA) in R (version 3.4.4):

$$Y_{\mathrm{ij}}=\mu +p_{i}+\varepsilon_{\mathrm{ij}}$$

where the response (*Y_ij_*) is a response matrix combining the nine leaf traits, *µ* is the intercept, *p* describes the genetic effect of source population, and ε residual variance. Significant differences among the source populations in the common garden provide evidence that trait differences between populations are primarily due to heritable genetic effects rather than nongenetic influences such as phenotypic plasticity. However, it is possible that some trait variation is a result of parental environment and we cannot rule out the influence of maternal effects.

#### 2.3.2 Seed production

To create populations for the field component of this study, plants were grown over two generations between January 2012 and November 2013 using the method outlined above. Controlled crosses were performed in common garden populations by rubbing flower heads from parental plants together to transfer pollen; as flower heads open over multiple days, we repeated this procedure for three days for each cross to maximize seed production. We did not pool reciprocal crosses and made sure only one direction of each cross was used in the field experiments. Families were generated by crosses within ancestral populations (Parental, P), between populations (F1 hybrids, F1), within F1s (F2 hybrids, F2), and between F1s and Parental (Backcrosses, BC). Few Backcross families were created between Dune‐Island F1s and Island Parental (BCID) due to limited flowering time overlap in the glasshouse, so we excluded this cross type from the experiment.

### 2.4 Field experiments

#### 2.4.1 Soil analysis

We collected soil from natural environments to characterize differences in soil composition between Dune headland and Island environments. Samples were analyzed by ALS environmental laboratories in Brisbane, Australia, and included measures of pH, fertility (carbon, phosphorous, potassium, nitrogen), metals (aluminum, titanium, iron) and salinity (bicarbonate, Electrical conductivity), and microorganisms (heterotrophic plate count, HPC).

#### 2.4.2 Reciprocal transplant experiment

To determine the role of divergent selection in driving these trait differences, we used phenotypic data collected from a reciprocal transplant experiment. The transplant was started on the 20 April 2014 in the Coffs Harbour at the locations of the initial seed collections and is described in detail elsewhere (Richards et al., 2016). Four plots were established in each of the three environments, each treated as individual blocks. Vegetation and litter were removed from the plots before planting. Seeds were glued to toothpicks using Selleys Quick fix supa glue gel and organized into a completely randomized block design across the four plots at each field location. One seed from each family was planted into the cells of 400 × 600 mm mesh grids with the seed placed ~2 mm below the soil surface. Plants were watered daily with 1 liter of water per block (equivalent to ~4 mm of rainfall) and covered with shade cloth for the first 21 days to induce germination. We included four individuals from each family, 15 families per cross type in each environment, leading to 60 individuals per cross type per environment. As our design included seeds from the wild populations and all possible crosses (except BCID) between the three populations, we planted 17 different cross types, leading to 1,020 seeds per environment and 3,060 seeds across the whole transplant.

The experiment was ended after 7 months. Five mature leaves were removed from each surviving transplanted synthetic cross plant. Additionally, we collected leaves from the resident natural populations, which represent reference populations at each experimental location. Five leaves from each of 31 established wild plants in the Dune environment, 54 plants in the Headland, and 32 plants in the Island populations were collected along transects that spanned the spatial distribution of each population.

#### 2.4.3 Statistical analysis

In the field component of this work, we apply a two‐step approach to investigate the adaptive significance of leaf shape variation in *S. lautus*. We first apply multivariate analysis of variance (MANOVA) to determine whether there is significant multivariate divergence in leaf shape between synthetic populations transplanted into three contrasting environments. We extract the discriminant functions from this MANOVA, the linear combination of traits associated with the greatest phenotypic divergence between transplanted populations. We then project these discriminant functions onto trait measurements from natural populations to determine whether the combination of traits which explain divergence between transplant populations also explain differences between the wild populations. Divergence in the same linear combination of traits in both transplant and natural populations is strong evidence that trait divergence between ecotypes in this system is driven by environmental factors. All analyses were performed using R statistical software (version 3.4.4).

First, to determine if multivariate leaf shape differed between transplant survivors in each environment, we conducted the following multivariate analysis of variance (MANOVA):

(1) $$Y_{\mathrm{ij}}=\mu +E_{i}+C_{j}+\varepsilon_{\mathrm{ijk}}$$

which combined the nine leaf morphology traits in a response matrix (*Y_ijk_*) and fixed effects environment (*E*), crosstype (*C*), and residual error (*ε*). We then calculated the divergence matrix (*D*) by extracting the sum squares of cross product matrices (SSCP) associated with the hypothesis of interest, environment, and the appropriate error term, the residual, in Equation (1), where:

(2) $$D=R^{-1}E$$

These matrices describe the ratio of explained (*E*) and unexplained (*R*) trait variance associated with the environment term and form the basis of the hypothesis test for a significant effect of environment on trait divergence, but can also be used to extract information on the linear combinations of traits that differ most between the three environments (Rencher, 2003). *R* and *E* are square, symmetrical matrices with the same number of dimensions as traits included in the analysis (9), and *D* is therefore a 9 × 9 matrix with a rank of 2, which is determined by the degrees of freedom of the environment term. Therefore, divergence between the three environments can be characterized by extracting the two first two eigenvectors from the *D* matrix: The first eigenvector (*d*_1_) is the linear combination of traits which varies the most between the three populations, followed by (*d*_2_) explaining the second axis of trait divergence (McGuigan, Chenoweth, & Blows, 2004; Rencher, 2003).

Second, these discriminant functions (*d*_1_, *d*_2_) were applied to phenotypic measurements from the natural populations to produce two new canonical variables describing a projection of transplant divergence onto natural population individual phenotypes to determine if the trait relationships distinguishing transplant populations also reflected differences between the wild populations. These two projections of natural phenotypes were then combined in the response matrix, *Y_ij_* to test whether they differed between environments using the multivariate analysis of variance:

(3) $$Y_{\mathrm{ij}}=\mu +E_{i}+\varepsilon_{\mathrm{ij}}$$

where a significant Environment term (*E*) would suggest the main axes of variation between transplant populations also separate natural populations on the basis of leaf shape.

## 3 RESULTS

### 3.1 Genetic basis of ecotype traits under common garden conditions

**Figure 2:**
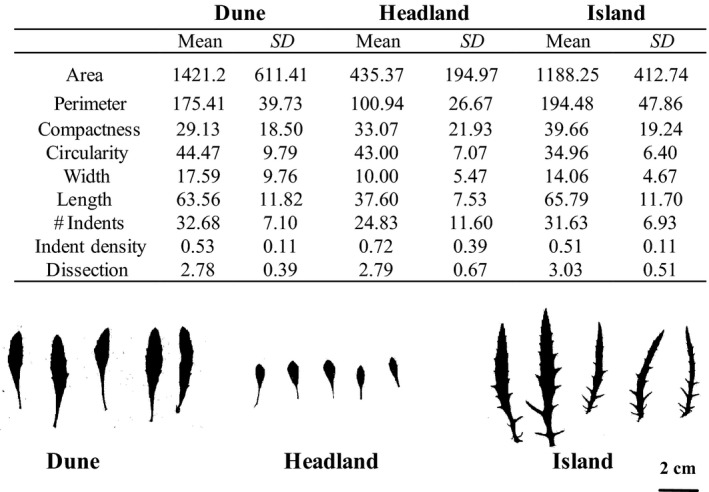
Leaf trait differences between populations of *Senecio lautus* grown in common garden experimental conditions. Archetypes of leaf shape from three ecotypes of *S*. lautus. Mean and standard deviation of leaf shape traits from the 28 Dune (D), 37 Headland (H), and 22 Island (I) plants grown in common garden from field‐collected seeds. Traits are defined as follows: area (mm^2^), circularity (% overlap with circle of equal area), compactness (perimeter: area), dissection (perimeter^2^/length), indent density (*n* indents/perimeter), length (mm), number indents (n), perimeter (mm), width (mm)

Divergence in leaf shape between wild populations is maintained when plants are grown under common garden conditions in the glasshouse, suggesting that genetic differentiation underpins leaf shape differentiation in this system. We found significant differences between ecotypes in all leaf shape traits except indent density (Figure 2) and a highly significant difference in the multivariate trait divergence between populations (Wilks *λ* = 0.144, *F*_approx_ = 13.81_18, 154_, *p *= <0.001). Leaves from the Headland population have smaller area and more serrated margins (more indents) than Dune and Island leaves. Island leaves are more compact (larger perimeter: area ratio) and are more dissect.

### 3.2 Soil analysis

Soil constituents reflect the strong differences between environments in the sand dunes, clay‐based headland, and guano‐rich Island soils (Table 1). Island soils had the highest concentration of nutrient, microorganism, and salts, and the lowest pH. Dune soil was at the other extreme, with the lowest counts across all soil constituents and the highest pH. Headland soils were slightly alkaline and intermediate in all measures except aluminum, which was the highest across the three environments.

**Table 1:** Soil constituents across Dune, Headland, and Island Environments at Coffs Harbour, NSW

Environment	Dune	Headland	Island
Soil components in natural populations			
pH	8.5	6.1	4.1
EC	37	79	284
Aluminum	490	18,100	6,690
Iron	2,040	10,900	24,400
Potassium	90	850	2,330
Phosphorus	60	240	3,940
Titanium	20	100	370
Total Nitrogen	70	5,360	10,500
Bicarbonate	0	3	230
Organic Carbon	0.03	6.4	7.25
HPC	45,000	630,000	2,400,000

Abbreviations: HPC: Heterotrophic plate count; EC: Electrical conductivity.

### 3.3 Leaf shape divergence

Of the 1,020 seeds planted out in each natural environment, 27 individuals survived to be sampled for leaf phenotypes in the Dune, 61 in the Headland, and 82 in the Island environments. This high mortality was primarily due to drought conditions that manifest for the second half of the field experiment. During this time, the field locations received less than 20% of the long‐term average rainfall leading to reduced sample sizes in the three environments. There was no significant effect of crosstype on leaf shape (Wilks *λ* = 0.155, *F*_approx_ = 1.09_(144, 592)_, *p* = 0.23), however survival analysis in a subset of this transplant experiment showed significant survival reduction in nonlocal genotypes (Richards & Ortiz‐Barrientos, 2016). Therefore, the nonsignificant cross type effect on leaf shape should be interpreted with caution based on the low power of this test, with low survival resulting in relatively low replication of the 17 crosstypes. Notably, nonnative individuals were present at the completion of the field experiments, suggesting that the differences in leaf shape between environments were not simply due to sole survivorship of local genotypes in each location. Leaf shapes of the transplant survivors showed a highly significant difference in multivariate leaf phenotypes between environments (Wilks *λ* = 0.375, *F*_approx_ = 5.3_(18, 146)_, *p* = <0.001). The major axis of divergence (*d*_1_) described 79.75% of phenotypic variance and was driven by changes in perimeter, length and number of indents between environments. The second axis of differentiation (*d*_2_) described the remaining 20.25% of variation and a combination of perimeter and number of indents. The main difference between these two discriminant functions is the removal of the size effect in *d*_2_ suggesting size and shape differentiate phenotypes along the first axis of variation, and only leaf shape traits separate them along the second axis of variation. (Table 2).

**Table 2:** Discriminant function loadings on the two axes of differentiation of transplant individuals between environments

Trait	*d*_1_	*d*_2_
Area	0.058	−0.292
Perimeter	−0.767	0.826
Compactness	−0.311	−0.203
Circularity	−0.063	−0.084
Width	−0.332	−0.199
Length	0.264	0.055
No of indents	0.314	−0.334
Indent density	0.117	0.151
Dissection	−0.127	−0.083
Eigenvalue	1.091	0.277
%	79.75	20.24

Note

Vector *d*_1_ is the first eigenvector of the matrix of phenotype divergence due to environmental effects, describes the combination of traits separating populations along this dimension and accounts for 79.75% of variation between sites. *d*_2_ is the second eigenvector describing the combination of traits separating populations along the second dimension of divergence and accounts for the remaining 20.25% of variation. Both vectors are comprised of leaf size and shape related traits, suggesting complex differentiation between environments.

**Figure 3:**
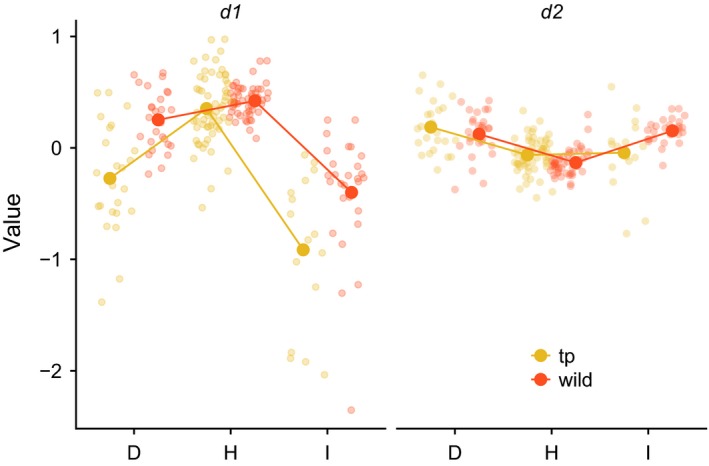
Projections of canonical discriminant functions on leaf traits in field transplants and natural populations of *Senecio lautus*. Predictions based on canonical discriminant functions extracted from transplant data. Transplant individuals are shown in yellow and illustrate divergence along both dimensions of divergence between environments *d*_1_ *, d*_2_. Discriminant functions separating transplant populations were projected into leaf shape data from natural populations shown in red to ask whether divergence in transplant populations reflected differences in the wild. Patterns were the similar along both axes of divergence which suggests that leaf shape differences found between ecotypes are the results of divergent ecological conditions

Projection of the discriminant function into the natural populations collected at the same sites predicted significant trait divergence between environments (Wilks *λ* = 0.206, *F*_approx_ = 59.49 _4, 220_, *p* = <0.001) suggesting divergence in transplant individuals reflects differences between the native populations. Visualization of the MANOVA results are depicted in Figure 3. The first axis of environmental *d*_1_, which accounted for ~80% of the variation in leaf shape among transplant plants, separates both transplant and natural populations in a similar pattern (Figure 3). Both *d*_1_ *and d*_2_ separate the transplant populations in each location and mirror patterns of divergence between the native populations.

The crossing design used to generate seeds for the field transplant ensured that the full range of heritable leaf shapes observed across the three ecotypes was planted within each environment. The pattern of divergence between the three transplant populations toward the natural population leaf shape in each environment (Figure 3) suggests that divergent natural selection is driving trait divergence among the transplanted plants from a shared mean toward mean phenotype of the local wild populations.

## 4 DISCUSSION

Here, we used artificial hybridization and field‐based transplant experiments to reveal the influence of divergent natural selection on phenotype evolution in three local adapted ecotypes in the *S. lautus* species complex. Leaf shape diverged toward the native character in each transplant location suggesting leaf shape divergence between these ecotypes is associated with adaptation to ecological differences faced by natural populations. The experimental recreation of phenotypic divergence in synthetic hybrid populations exposed to divergent ecological conditions provides direct evidence that ecologically derived natural selection drives leaf shape evolution in this system.

Common garden experiments revealed heritable differences across all leaf shape traits. Dune and Island ecotypes are similar in many aspects of leaf shape; however, Island leaves are more dissect, while the Headland phenotype is distinct across many traits and is characterized by smaller leaves with more serrated margins. These patterns also form the basis for the main axes of divergence in field transplant individuals, which were characterized by differences in the relationship between leaf traits describing size (perimeter, width, length) and shape (compactness, number of indents, indent density). We do not directly estimate heritability (*H* ^2^) in these experiments, however the retention of leaf shape in the absence of natural environmental conditions combined with heritability estimates drawn from controlled environment experiments in other *S. lautus* populations (Walter et al., 2018), and results from several previous taxonomic studies (Ali, 1966; Ornduff, 1964; Radford et al., 2004) support the premise that phenotypic differences between natural populations have evolved as a result of divergent selection on traits that confer an adaptive advantage in each environment.

In this work, we assess the significance of leaf shape as an adaptive trait through a comparative field experiment across three distinct environments. Transplant sites are located in close proximity and as such share many similarities in atmospheric environment, however soil analysis reveals two main environmental classes in which these sites differ: soil fertility and water availability. While it is difficult to determine whether the function of a particular leaf phenotype is itself adaptive, or whether the observed patterns reflect selection on an unmeasured but correlated trait (Nagy, 1997), there are some consistent trends between leaf shape and environmental conditions (Givnish, 1987; Nicotra et al., 2011) which suggest that leaf shape is associated with adaptation to ecological conditions. Reduction in leaf size is associated with solar exposure and temperature (Ackerly, Knight, Weiss, Barton, & Starmer, 2002), elevated salinity, and nutrient limitation (McDonald, Fonseca, Overton, & Westoby, 2003). Aluminum toxicity is also associated with stunted growth and reduced leaf expansion (Kidd & Proctor, 2000), an effect that is compounded in high pH soils. It is therefore likely that the smaller leafs found on plants in the headland environment are due to the elevated aluminum, high pH, and high salinity found in the headland soil environment and suggests that tolerance to these soil components may be important for local adaptation in this environment.

In reduced salinity and elevated nutrient environments, larger leaves might be expected, a pattern reflected in the results from the higher nutrient Island environment, and the reduced salinity Dune environment. As general trends in leaf shape suggest that leaf size is related to the major differences between these environments (fertility and moisture availability), it is likely that leaf shape divergence in this system is associated with local adaptation, with smaller leaves associated with aluminum toxicity and water stress and larger leaves associated with high soil fertility.

It is typically difficult to determine how selective pressure has shaped phenotypic variation in wild populations, as divergent selection has already removed many low fitness alleles from the population, and viability selection operating early in life further reduces the range of phenotypes observed in the population each generation (Mojica & Kelly, 2010; Polak & Tomkins, 2013). The utility of a synthetic hybrid population in these experiments is to break down trait correlations through recombination, thus generating a range of phenotypes outside those naturally found within the wild populations. Using this approach, we can determine how selection may shape variation across a wider part of phenotypic space than is possible in unmanipulated populations (Lexer et al., 2003). Nonetheless, demonstrating direct, rather than correlated, selection is a general problem in evolutionary biology as the characterization of an individuals' phenotype will almost always by incomplete. The nonrandom association of phenotype and environment observed in these experiments suggests that some aspect of leaf shape is associated with elevated fitness, either directly or as a result of strong pleiotropy with some “hidden” trait under selection.

Divergence of leaf shape phenotype in transplanted populations toward the character of the natural populations in each location is evidence that these phenotypes are associated with adaptations, and that ecotype distinctions are maintained by selection. If gene flow or migration were to occur between populations, selection should remove or substantially reduce fitness in those immigrants with different phenotypes, making the link between adaptation to divergent ecological conditions and the beginning of reproductive isolation. The substantial shift in phenotypes observed here within a single generation suggests that selection against immigrants could be strong in this system, a pattern observed in other studies on these populations (Richards & Ortiz‐Barrientos, 2016; Richards et al., 2016). These results are consistent with the definitions of the early proponents of the ecotype, where heritable adaptations to divergent ecological conditions do not simply produce morphological variants within a species distribution, but may be a step toward the generation of new species.

## CONFLICT OF INTEREST

None Declared.

## AUTHOR CONTRIBUTIONS

TJR, KM, DOB designed the research. TJR performed the experiments, fieldwork, and data analysis. TJR, KM, DOB wrote the manuscript.

## Data Availability

Data and R code is uploaded to the Dryad Database. Provisional DOI: https://doi.org/10.5061/dryad.88j4772.
