# Supplementary material for: Natural selection drives leaf divergence in experimental populations of Senecio lautus under natural conditions
Source: Ecol Evol. 2019 Jun 11;9(12):6959–67. doi: 10.1002/ece3.5263 (PMC6662321; doi:10.1002/ece3.5263)
Supplement: Supplementary file 1 [file ECE3-9-6959-s001.docx]

TABLE S1 – LAMINA trait values

| Trait | Corresponding LAMINA metric | Definition |
| --- | --- | --- |
| Area | Area2 | Area (mm2) of leaf ignoring cavities |
| Perimeter | Perimeter2 | Perimeter (excluding cavities) mm |
| Compactness | Squared perimeter2/Area2 | Perimeter:Area ratio |
| Circularity | Circularity | Overlap of leaf outline and circle of same area |
| Width | Horizontal size centre | Leaf width at widest point (mm) |
| Length | Vertical size centre | Leaf length at longest point (mm) |
| # Indents | Number of indents | Number of serrations/indents |
| Indent density | Number of indents/vertical size centre | Number of serrations per mm of leaf length |
| dissection | Perimeter2/vertical size centre | Perimeter:Length ratio |
